# Discovery of Putative Herbicide Resistance Genes and Its Regulatory Network in Chickpea Using Transcriptome Sequencing

**DOI:** 10.3389/fpls.2017.00958

**Published:** 2017-06-07

**Authors:** Mir A. Iquebal, Khela R. Soren, Priyanka Gangwar, P. S. Shanmugavadivel, K. Aravind, Deepak Singla, Sarika Jaiswal, Rahul S. Jasrotia, Sushil K. Chaturvedi, Narendra P. Singh, Rajeev K. Varshney, Anil Rai, Dinesh Kumar

**Affiliations:** ^1^Centre for Agricultural Bioinformatics, Indian Agricultural Statistics Research Institute (ICAR)New Delhi, India; ^2^Division of Plant Biotechnology, Indian Institute of Pulses Research (ICAR)Kanpur, India; ^3^Genetic Gains, International Crops Research Institute for the Semi-Arid TropicsPatancheru, India

**Keywords:** chickpea, differentially expressed genes, gene regulatory network, herbicide, Imazethapyr, molecular markers, transcriptome

## Abstract

**Background:** Chickpea (*Cicer arietinum* L.) contributes 75% of total pulse production. Being cheaper than animal protein, makes it important in dietary requirement of developing countries. Weed not only competes with chickpea resulting into drastic yield reduction but also creates problem of harboring fungi, bacterial diseases and insect pests. Chemical approach having new herbicide discovery has constraint of limited lead molecule options, statutory regulations and environmental clearance. Through genetic approach, transgenic herbicide tolerant crop has given successful result but led to serious concern over ecological safety thus non-transgenic approach like marker assisted selection is desirable. Since large variability in tolerance limit of herbicide already exists in chickpea varieties, thus the genes offering herbicide tolerance can be introgressed in variety improvement programme. Transcriptome studies can discover such associated key genes with herbicide tolerance in chickpea.

**Results:** This is first transcriptomic studies of chickpea or even any legume crop using two herbicide susceptible and tolerant genotypes exposed to imidazoline (Imazethapyr). Approximately 90 million paired-end reads generated from four samples were processed and assembled into 30,803 contigs using reference based assembly. We report 6,310 differentially expressed genes (DEGs), of which 3,037 were regulated by 980 miRNAs, 1,528 transcription factors associated with 897 DEGs, 47 Hub proteins, 3,540 putative Simple Sequence Repeat-Functional Domain Marker (SSR-FDM), 13,778 genic Single Nucleotide Polymorphism (SNP) putative markers and 1,174 Indels. Randomly selected 20 DEGs were validated using qPCR. Pathway analysis suggested that xenobiotic degradation related gene, glutathione S-transferase (GST) were only up-regulated in presence of herbicide. Down-regulation of DNA replication genes and up-regulation of abscisic acid pathway genes were observed. Study further reveals the role of cytochrome P450, xyloglucan endotransglucosylase/hydrolase, glutamate dehydrogenase, methyl crotonoyl carboxylase and of thaumatin-like genes in herbicide resistance.

**Conclusion:** Reported DEGs can be used as genomic resource for future discovery of candidate genes associated with herbicide tolerance. Reported markers can be used for future association studies in order to develop marker assisted selection (MAS) for refinement. In endeavor of chickpea variety development programme, these findings can be of immense use in improving productivity of chickpea germplasm.

## Introduction

Chickpea (*Cicer arietinum* L.), also known as Bengal Gram is one of the major pulses contributing over 75% of the total production of pulses in the world. It is a major and cheap source of protein as compared to animal protein, thus, important for nutritional security in the developing countries. It is grown and consumed in the large quantities in South East Asia, India, Middle East and Mediterranean countries (Varshney et al., 2013). The global chickpea production is about 13.1 million tons from an area of 13.54 million hectares. India is the largest producer contributing about 67.3% of the total world production (Faostat, 2013).

Weeds are one of the important constraints for chickpea production, as it competes for water, nutrients, light and space. In the initial stage of growth, chickpea has an open canopy architecture and low stature, which reduces its ability to compete with weeds (Amor and Francisco, 1987; Knights, 1991). Weeds also increase expenditure on labor, equipment and chemicals for their control. Even many weeds harbor fungal/bacterial diseases and insects/pests which further adversely affects productivity. For chickpea, first 6 weeks of crop growth are more critical with respect to weed competition. During this stage of crop growth, weeds can reduce chickpea pod dry-mass up to 40% (Tripathi, 1967) and yield loss up to 95% (Ali, 1989). However, effective control of weeds can increase the yield of chickpea from 17 to 105 % (Taran et al., 2013).

Weed can be managed more effectively by developing herbicide tolerance crop varieties. New herbicide are limited in number having constraints of regulatory clearance (Tan et al., 2005). Application of herbicide can lead to adverse human health due to chemical residues in chickpea. Development of herbicide resistant crop varieties can maximize long term benefit and reduce weed shift to difficult-to-control and herbicide resistance weeds. In three decades with expense of billion of dollars, only few transgenic herbicide traits could be developed (Green and Owen, 2010). Herbicide tolerant crop varieties have efficient enzymatic system which degrades and/or detoxify herbicide more efficiently thus they also accumulate less herbicide (Botterman and Leemans, 1988).

Among the genetic approaches, both transgenic and non-transgenic approaches have been attempted for the development of herbicide resistant varieties of plants. Though the transgenic approach has been reported to be successful in many agricultural crops like corn, soybean, cotton etc. (Devine, 2005) but this approach has several limitations like long term/high costs research and development cost, cost of regularity clearance and global trade issues of genetically modified organisms (GMOs) etc. Beside these, ecological and environmental issues make it further compounded (Kwon and Kim, 2001). On the other hand, new herbicide use has issues of registration, environmental clearance and impact on existing herbicides (Devine, 2005). Recent prohibition of herbicide (glyphosate) resistant crop for example wheat and sugarbeet by Latin America, Russia, Mexico, Netherlands and many other countries further necessitates some long term sustainable solution (Duke, 2005). Further, in case of genetically modified herbicide-resistant crops (GM-HRC) of legume, a major concern is that the transgene may get introgressed into related weeds (Singh and Yadav, 2012). Though, gene responsible for herbicide tolerance has been reported on 1984 (Chaleff and Mauvais, 1984), but very recently a single gene associated with herbicide tolerance, acetohydroxyacid synthase (*AHAS*) (with two homologous with 80% similarity) having a point mutation (C675 to T675) leading to one amino acid (Ala205 to Val205) change has been reported in chickpea. This development is very promising for the herbicide resistant variety development of chickpea (Thompson and Tar'an, 2014). For introgressing more such genes, a holistic transcriptome based candidate gene/key pathways involved in herbicide tolerance discovery is desirable. Such genes can be of immense use in variety improvement programme for trait, herbicide tolerance of chickpea. This would be more effective strategy to control weeds without compromising chickpea productivity.

In order to discover herbicide tolerant genes, holistic approach is needed using susceptible and tolerant genotypes associated with herbicide tolerance. Such approach has been reported in many other crops like maize, wheat, sorghum, rice, oilseed (*Brassica napus* L.), and sunflower (Rubiales and Fernández-Aparicio, 2011). Since, large variability in tolerance limit of herbicide is already reported in chickpea varieties (Gaur et al., 2013), thus, it is expected that some of the genes offering herbicide tolerance can be introgressed from high tolerant donor varieties to high yielding sensitive varieties in chickpea variety improvement programme.

Genome based SNP and Indel information in chickpea has been reported (Doddamani et al., 2015) but transcriptome based is yet to be reported. Moreover, these are also categorized in terms of various parameters of SNP classification but none of them are related to herbicide tolerance, which is an agriculturally important trait.

The present work aims at identification of genes associated with herbicide tolerance in chickpea using susceptible and tolerant genotype by transcriptomic approach. It also aims at prediction of microRNA binding sites, transcription factors (TFs), gene regulatory network (GRN) depicting hub protein genes along with the genic region putative markers viz., SSRs, SNPs and Indels.

## Methods

### Plant material

The two contrasting chickpea genotypes, herbicide tolerant (HT), ICC1205 and herbicide susceptible (HS), ICC5434 identified and screened for post emergence herbicide, Imazethapyr (Gaur et al., 2013) were studied for the genome wide differential expression of transcripts. The surface-sterilized chickpea seeds of both the genotypes were germinated on earthen pot containing vermiculite: soil (1:1) mixture kept under controlled environmental chamber. The whole experiment was conducted in a temperature-controlled puff chamber with a day/night temperature regime of 28/23°C, respectively, with 16 h of photoperiod. The fully grown seedling of 25 days after sowing was treated uniformly by spraying with Imazethapyr (Pursuit: BASF) at the rate of 750 ml ha^−1^ as per the label recommendation. The young leaf tissue samples were harvested aseptically before and after 16–48 h of treatments and immediately frozen in liquid nitrogen at −80°C for total RNA isolation. Across sample variability minimization was done by sample pooling approach of 10 biological replicates of each genotype (Zou et al., 2016). The young leaf tissues from different chickpea plants were pooled before processing RNA extraction.

### Total RNA isolation, library preparation, and transcriptome sequencing

Imazethapyr, the weedicide which mainly kills the growing tips (apical meristem and young leaves) of the plants and sometimes it leads to death of highly sensitive genotypes. Here, we used young leaf tissue for characterization of the leaf-specific transcriptome. Total RNA was isolated using RNeasy mini kit (QIAGEN, Hilden, Germany) and quantified by fluorescence based Quant-iTTM Ribogreen RNA Assay Kit. It detected total RNA mass range between 2,600 and 224,000 ng (130–11,200 ng/μl). The integrity of total RNA was determined using Bioanalyzer and the RIN (RNA Integrity Number) value measured above 6.6 were subsequently used.

Illumina sequencing was performed using the HiSeq™ 2,000 platform according to the manufacturer's instructions (Illumina, San Diego, CA). The sequencing data were deposited to National Center for Biotechnology Information (NCBI) (BioProject accessions: PRJNA306813; BioSample accessions: SAMN04364319, SAMN04364320, SAMN04364321, and SAMN04364322).

### Pre-processing of transcriptome datasets

In this study, Illumina paired-end reads were generated using RNA of two chickpea genotypes, namely, ICC1205, and ICC5434. In case of ICC5434 (HS) genotype, approximately 22 million and 24 million paired-end reads were generated before and after herbicide treatment, respectively. Similarly, for ICC1205 (HT), approximately 22 million and 20 million paired-end reads were generated before and after herbicide treatment, respectively. All the raw reads were pre-processed for adaptor contamination with parameters read length ≤ 36, poor quality ≤ 3, and HEADCROP:10 bases using trimmomatic software (Bolger et al., 2014). Subsequently pre-processed data were used for transcriptome assembly, followed by DEGs identification in four combinations, namely, HS-Control vs. HS-Exposed (H[SC][SE]), HS-Control vs. HT-Control (H[SC][TC]), HT-Control vs. HT-Exposed (H[TC][TE]) and HS-Exposed vs. HT-Exposed (H[SE][TE]), TFs and miRNA binding site prediction, molecular markers' mining (SSR, SNP, and Indels).

### Transcriptome assembly

Processed high-quality reads were assembled using Trinity and Tophat software (Haas et al., 2013; Kim et al., 2013). For reference-based assembly, genome of chickpea (*desi* type) was downloaded from chickpea genome analysis project (Parween et al., 2015). All the four datasets were assembled separately with reference to this genome using Tophat software. Initially, the processed reads were mapped onto the reference genome and the individual annotation file were created using Cufflinks (Trapnell et al., 2010). Finally, all the annotation files were merged using Cuffmerge (Trapnell et al., 2010) to get a single assembly file. The aim of *de novo* assembly of unmapped reads was to search for the genes not observed in reference-based assembly (called extra-genes). Therefore, all unmapped paired-end reads (3,692) were pooled from each sample and Trinity was used for assembly (Palmieri et al., 2012; Kazemian et al., 2015). Finally, all assembled contigs from *de novo* assembly were used as input for blast search against a non-redundant database of NCBI.

### Identification of differential expressed genes (DEGs) and transcription factors

In order to quantify the gene expression, count-based method, i.e., HTSeq (Anders et al., 2014) was used. Finally, the transcript counts were used for pairwise differential gene expression analysis using edgeR package (Robinson et al., 2010). A cut-off value of log2 ratio ±2 and *q*-value 0.05 were used to filter out the significant transcripts in each case.

For the construction of gene regulatory network, a log2 value ± 5, *q*-value 0.05 and logCPM ≥ 5 for tolerant (control vs. exposed) and susceptible (control vs. exposed) were used followed by normalization of expression value. However, in case of susceptible control vs. tolerant control and susceptible-exposed vs. tolerant-exposed, a log2 value ± 2, *q*-value 0.05 and logCPM ≥ 5 were used followed by normalization of expression value. For correlation analysis and network construction and its visualization, expression correlation plugin (http://apps.cytoscape.org/apps/expressioncorrelation) and Cytoscape v.3.2.1 software were used (Shannon et al., 2003). Further, transcription factors were identified in *C. arietinum*, Transcriptome Assembly version 2 (CaTA v2, available at http://data.comparative-legumes.org/transcriptomes/cicar/lista_cicar-201201), was compared to plant-specific transcription factor database PlnTFDB (Pérez-Rodríguez et al., 2010) using BLASTX search with the stringency of *E*-value 1e-06 (Moriya et al., 2007).

### Functional annotation and prediction of microrna controlled genes

The differentially expressed genes were further subjected to functional annotation using BLASTX similarity search against NCBI non-redundant database at *E*-value 1e-5 (Shannon et al., 2003). Also, pathway assignment was performed using KASS server against the Kyoto Encyclopedia of Genes and Genomes (KEGG) database (Altschul et al., 1990). A BLASTX query using bi-directional search was used with respect to dicot plant gene family. Functional categorization and domain search were performed using GO ontology and IPRscan module of BLAST2GO software (Conesa and Götz, 2008). Additionally, miRNAs, which are the important post-transcriptional gene regulator in response to nutritional, biotic, and abiotic stresses were predicted *in silico* (Sunkar et al., 2006; Unver et al., 2010; Zhang et al., 2013). For this, all mature miRNAs of Fabaceae family (1,545) from miRBase were used to search their target in chickpea differentially expressed genes using psRNATarget webserver (Griffiths-Jones et al., 2006; Dai and Zhao, 2011).

### Marker discovery

Both, assembled transcripts and differentially expressed transcripts were used to identify SSRs as putative marker. MISA (http://pgrc.ipk-gatersleben.de/misa/) tool was used for detection of microsatellites from mono- to hexa-nucleotides with a minimum repeat number of eight for di-nucleotides and five for others. Primer3 software (Koressaar and Remm, 2007; Untergasser et al., 2012) was used for designing primers for identified SSR markers with parameters such as annealing temperature (Tm) min:57, optimal:60°C, and maximum:63°C, primer size min:15, optimal:18, and maximum:21 oligo-nucleotides (Rozen and Skaletsky, 2000).

For SNP and Indel discovery, only whole transcriptome assembly (pooled reference), was used. The sorted alignment files from each sample were used to produce a bcf file using Samtools (Li et al., 2009). The bcftool was used to filter SNPs at *p*-value <0.05. The potential SNPs were identified using read depth (d) ≥ 10, quality depth (Q) ≥ 30, MQ (minimum root mean square mapping quality) ≥ 40 and flanking sequence length (l) = 50. The snpEff was used for analyzing the impact of identified SNP's in transcriptome (Cingolani et al., 2012).

### Validation and expression analysis by RT-PCR

The first strand cDNA synthesis was done using SuperScript™IIIRT (Invitrogen life technology) as per manufacture's protocol. qPCR primers were designed using randomly selected 20 transcripts using Primer 3 software (Koressaar and Remm, 2007; Untergasser et al., 2012). Chickpea *GAPHD* (Glyceraldehyde-3-phosphate dehydrogenase) gene was used as housekeeping gene for normalization. PCR was performed using SYBR Green (7500 Applied Bio systems Foster, CA, USA) using standard 40 cycles along with melt curve step. PCR conditions were standardized to obtain amplification in linear relationship. Each reaction was carried out in triplicates and ΔΔC_T_ values were obtained.

## Results and discussion

### Transcriptome assembly

Data from the susceptible and tolerant genotypes of herbicide tolerance transcriptomic (more than 90 million paired-end reads) was obtained successfully. After the pre-processing and quality check, 72.11 million paired-end reads (Table 1) were finalized for further analysis. For herbicide-tolerant genotype, ICC1205, 27371448, and 34213538 paired-end reads were obtained for control and treated, respectively. Similarly, after the pre-processing and quality check of data for herbicide susceptible genotype, i.e., ICC5434, 42974090, and 39668970 paired-end reads were obtained for control and treated, respectively. Reference based assembly using Tophat resulted in the generation of 30,803 contigs with the largest contig of length 14,744 nucleotides. Further, 16,645 contigs with length >1,000 and with N50 value of 1,706 was obtained using reference-based assembly (Table 2). The assembly of unmapped reads resulted in 42 transcripts (length >200 nucleotides) which were missed in reference-based assembly. Further, Blast analysis gave 31 hits out of which 18 were from leguminous family (Additional File 1). The mapping revealed approximately 55.1–79.8% reads on the reference genome. A multiple site mapping of 5.5 and 4.3% was also observed in control and treated sample of ICC1205 (HT) genotype, respectively while in case of ICC5434 (HS) genotype, it was found to be 4.9 and 4.4%, respectively (Table 3).

**Table 1:** Chickpea transcriptome dataset from two extreme genotypes, i.e., susceptible (ICC5434) and tolerant (ICC1205).

**Genotype**	**Type**	**Class**	**Raw reads**	**High quality reads**
ICC1205	Tolerant	Control	45,687,858	27,371,448
ICC1205	Tolerant	Exposed	41,865,804	34,213,538
ICC5434	Susceptible	Control	49,473,436	42,974,090
ICC5434	Susceptible	Exposed	44,822,720	39,668,970

**Table 2:** Assembly statistics of chickpea transcriptome.

**Type**	**Tophat**
Number of contigs	30,803
Contig with size more than 1,000 bp	16,645
Largest contig	14,744
N50	1,706
L50	7,746
GC (%)	41.18

**Table 3:** Alignment statistics of chickpea transcriptome.

	ICC1205 (HT^*^)		ICC5434 (HS^*^)	
**Reads**	**Control (%)**	**Exposed (%)**	**Control (%)**	**Exposed (%)**
Mapped	55.1	72	79.8	77.5
Concordant	48.8	64	71	69.1
Disconcordant	6.5	6.1	6.3	6.3
Multi mapped	5.5	4.3	4.9	4.4
Unmapped	44.9	28	20.2	22.5

* HS, Herbicide Susceptible;

* *HT, Herbicide tolerant*.

### Identification of differential expressed genes (DEGs)

**Figure 1:**
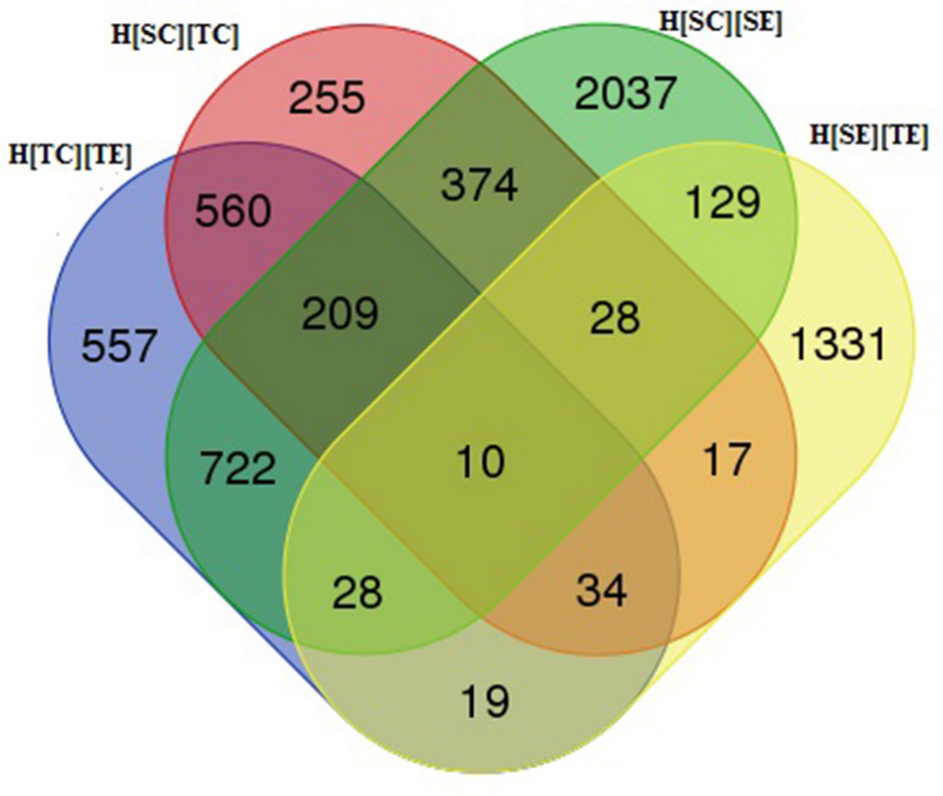
Venn diagram of differentially expressed genes in chickpea genotypes.

In order to delineate the herbicide resistance mechanism in chickpea transcriptome, four sets of comparison was made, viz., H[SC][SE], H[SC][TC], H[TC][TE], and H[SE][TE]. The alignment results of each sample were used for differential gene expression analysis by HTSeq count software. It was observed that 1,487 transcripts were differentially expressed from susceptible (ICC5434) to tolerant (ICC1205) genotype under control condition (Table 4, Additional File 2). In the present study, 3,537 transcripts showed a significant change in expression level after herbicide treatment on susceptible plants (Table 4, Additional File 2). A total of 2,139 transcripts were differentially expressed after herbicide exposure in ICC1205 (HT) genotype and 1,596 transcripts were differentially expressed after herbicide exposure to both tolerant and susceptible genotypes (Table 4, Additional File 2). The common and unique DEGs of each set is shown in the Venn diagram (Figure 1). This comparison reveals a total of 1,331 unique DEGs related to herbicide tolerance in chickpea.

**Table 4:** Differentially expressed genes count.

**DEG**	**Total (Blast hit)**	**Up**	**Down**
H[SC][SE]	3,537 (3,517)	1,937	1,600
H[SC][TC]	1,487 (1,474)	447	1,040
H[TC][TE]	2,139 (2,123)	1,412	727
H[SE][TE]	1,596 (1,591)	891	705

Interestingly, we did not find any significant change in expression of acetolactate synthase (*ALS*). It is also known as acetohydroxy acid synthase (*AHAS*) which is a well-reported herbicide resistant gene. Since herbicide imazethapyr is an inhibitor of *ALS*, thus it is aptly clear from our study that the herbicide resistance does not operate through differential expression of this gene. In other crops, this ALS gene is reported to be involved in herbicide resistance due to mutation rather than differential expression. This gene catalyzes the first step in the synthesis of the branched-chain amino acids viz., valine, leucine, and isoleucine (Chaleff and Mauvais, 1984; McCourt et al., 2006). Mutation in herbicide resistance is similar to at least 17 amino acid residues which are reported in bacteria, fungi, or plants causing such resistance (Duggleby et al., 2008). One mutant with high level (>10-fold) of resistance to imidazolinone was identified in *C. reticulatum* (Toker et al., 2012). Similar mutation has also been reported in other crops like tobacco and cotton (Subramanian et al., 1991).

### Identification of transcription factors and miRNA controlled DEGs

A total of 2,876 transcription factors in 1,024 transcripts from complete transcriptome were identified (Table 5). However, only 1,528 transcription factors were predicted to have binding site over 897 differently expressed genes (Additional File 3). Number of TF is more than number of DEGs, reflecting one gene having multiple TFs (Wagner, 1999) and it may be also due to overlapping sequence (Ji et al., 2012) and computational stringency (Boeva, 2016). Computational studies indicate that TFs and miRNAs form a complex regulatory network with their respective target genes. These two regulatory circuits are pivotal in coordinating transcriptional and post-transcriptional control of targeted genes (Cui et al., 2007). Our enlisted TF genes can be targeted for future SNP discovery. Such SNP in TF gene can change the agriculturally important traits in the crop, for example DELLA protein negative regulator causing dwarfism in oil plant (Rahman et al., 2015). Another example is *Prunus*, where, TFs control many agriculturally important traits like the flowering, fruit quality, and biotic and abiotic stress resistance (Bianchi et al., 2015).

**Table 5:** Predicted transcription factors in transcriptome assembly and DE Unigenes.

**Transcription Factors (TFs)**	**Whole transcriptome assembly**	**DE Unigenes**
Total transcripts	30,803	6310
Total number of TF predicted	2,876	1528
Number of transcripts having binding sites for TF	1,024	897

In this study, 3,037 genes regulated by 980 miRNAs (Additional File 4) were identified. Out of these, 411 genes regulated by 179 miRNAs were involved in defense mechanism. Likewise, 101 miRNA targets in 294 transcripts were found to be involved in biotic and abiotic stress. Some of these might be regulating herbicide resistance which needs further investigation. Enlisting such microRNAs can be a future genomic resource for further research/validation, especially to get miRNA for silencing technology. Such gene silencing of miRNA which controls DEG has already been reported in other crops (Li et al., 2014).

## Functional annotation of DEGs

### Expression analysis of susceptible vs. tolerant genotype without exposure: H[SC][TC]

The GO term analysis of biological process resulted in the identification of genes related to sepal, petal formation, ovule development and root hair elongation. Molecular function analysis identified five transcripts for solute/proton anti-porter and one for calcium/cation anti-porter activity. The KEGG pathway analysis suggested 1,487 differentially expressed transcripts even without herbicide treatment. Among the DEGs, ten encoded for amino acid biosynthesis, three genes for *CYP450*, and six genes encoded for *ABC transporter* genes. We also found 32 genes for signal transduction of plant hormone. We observed that in the tolerant plant, genes involved in photosynthesis showed an increase in expression. Similarly, genes for lysine, phenylalanine, threonine, and glutamine synthesis were found to be up-regulated in the tolerant plant. However, tyrosine and tryptophan synthesis pathway genes were found to be down-regulated in susceptible plants.

Gene co-expression analysis of 147 highly significant genes (logFC ± 2, logCPM ≥ 5, and FDR ≤ 0.05) resulted in top 28 genes with largest difference (Additional Files 5, 6). From these genes, we selected a highly expressed gene (xyloglucan endotransglucosylase/hydrolase) involved in cell wall construction (Kern et al., 2005). Further analysis of connected nodes with this gene resulted in 92 positively correlated and 49 negatively correlated genes (Supplementary Figure 1). Of these 92 positively correlated genes, three were transporters, one ribosomal protein, one mitogen-activated protein kinase (*MAPK*), and two genes were related to auxin hormone signaling process. Overexpression of these genes in the tolerant plant was found to be positively correlated with their biological functions like cell growth and divisions (Krysan et al., 2002). Similarly, in case of down-regulated genes, we observed that genes encode for histone protein were down-regulated which are involved in keeping the DNA in condensed form.

### Expression analysis of susceptible vs. tolerant genotype with herbicide exposure: H[SE][TE]

A total of 1,596 DEGs having 891 up and 705 down-regulated genes were found in this set. Blast analysis revealed 1,591 DEGs having similarity with other genes. Out of these 1,596 genes, 1,331 were found to be unique to this set. Nineteen genes were common to H[TC][TE] and H[SE][TE]. Further, 28 genes were common to H[SC][SE], H[SE][TE] and H[TC][TE] (Additional File 2).

Maximum hits were found with *C. arietinum*, followed by *Medicago truncatula* and *Glycine Max* (Supplementary Figure 2). Biological process were found to be associated with oxidation-reduction process, protein phosphorylation and peptidyl-tyrosine phosphorylation in 173, 78, and 44 transcripts, respectively. Analysis of GRN of 136 highly significant genes (logFC ≥ +-2; FDR ≤ 0.05; logCP ≥ 5) showed 129 and 7 as up- and downregulated, respectively (Additional Files 5, 6). We found differential expression of some well reported herbicide tolerance associated genes in this set of comparison.

Among 1,331 unique differentially expressed genes in the set H[SE][TE], we observed high expression of cytochrome P450 in tolerant genotype in our study which is due to its role in enhancing the rate of herbicide metabolism (Vila-Aiub et al., 2005). Similarly, higher expression of xyloglucan endotransglucosylase/hydrolase is reported to be associated with abiotic stress in hot pepper (Choi et al., 2011) and tea (Jayaswall et al., 2016). Well known herbicide detoxifying enzyme, glutamate dehydrogenase (Block et al., 1987) was found to be highly expressed in resistant genotype as already reported in tobacco (Nolte et al., 2004). We found very high expression of isoform of methyl crotonoyl carboxylase gene, such isoform based sensitivity and tolerance mechanism has been reported in biotypes of forage crop (Prado et al., 2000).

Among 19 DEGs in the set H[SE][TE] overlapping with H[TC][TE], key genes controlling herbicide tolerance pathways were found. Pectate lyase gene is well known for its role in herbicide resistance by controlling plant polysachharide composition which affects stress resistance (Liang et al., 2017). Protein strubbelig-receptor family 3 genes play role as sensor for herbicide, mediating signal of inter and intracellular ABA pathway regulating herbicide stress (Osakabe et al., 2013). Aquaporin nip5-1 gene controls the pore size thus critical in herbicide tolerance (Gupta, 2013). Cytochrome P450 having role in herbicide metabolism was also observed in this set of genes (Vila-Aiub et al., 2005).

Among 28 DEGs in the set H[SE][TE] overlapping with H[TC][TE] and H[SC][SE], genes associated with herbicide tolerance were found. Della gene has role in regulation of GA signaling by suppressing stress response pathways (Nakamura and Asami, 2014). This gene is associated with growth inhibition of weed (Heuer et al., 2016). Peroxidase 12-like gene has role in herbicide resistance by regulating jasmonic acid biosynthesis pathway. This gene is also involved in herbicide detoxifying reaction as well as abiotic stress tolerance (Abdeen and Miki, 2009; Dou et al., 2016). Pectinesterase inhibitor 34 is involved in fine-tuning cell wall remodeling processes in abiotic stress (Wang et al., 2016).

### Effect of herbicide exposure on susceptible genotype: H[SC][SE]

The GO term analysis identified 1006 transcripts for enzymatic activity out of which, 367 were hydrolases, 36 were isomerases, 430 were oxidoreductases, 82 were ligases, and 91 were lyases. The analysis of identified BLAST hit revealed the maximum number of hits from *C. arietinum*. Similarly, biological process resulted in a large group of transcripts associated metabolic process and transport (Supplementary Figure 3). The KEGG pathway analysis suggested that out of the 3,537 differentially expressed transcripts, 30 genes encode for amino acid biosynthesis, 16 genes for *CYP450*, and 11 genes encode for *ABC transporter* genes. We also found 12 and 64 genes that control the DNA replication and plant hormone signal transduction process, respectively. We also observed that auxin and cytokinin receptor genes were down-regulated, as these are responsible for cell growth and its division after herbicide exposure. Similarly, *cyclin D3*, and *TCH4* genes involved in brassinosteroid pathway were also down-regulated. However, a gene involved in the Abscisic acid pathway was found to be up-regulated which leads to stomatal closure (Zabalza et al., 2004). We observed that 2,166 genes exclusively changed their expression due to herbicide exposure. We observed that a gene from DNA polymerase (alpha subunit) was also down-regulated after herbicide exposure on susceptible plants. However, we have not observed any significant change in expression of herbicide target (*ALS*) gene. Analysis of gene regulatory network of 168 most significant genes (logFC ± 5, logCPM ≥ 5, and FDR ≤ 0.05) resulted in the identification of *GST* gene with the highest degree linked to 68 other genes of which 10 genes were downregulated and 58 were upregulated (Additional Files 5, 6). Among downregulated genes, one gene belongs to photosynthesis process, one gene for plant development and defense-related process (Folgado et al., 2013). Out of 68 overexpressed genes, UDP-glucosyltransferase, which also play an important role in xenobiotic metabolism (Punja, 2005; Yuan et al., 2007) was found. Similarly, the gene of ethylene responsive transcription factor which binds to ethylene receptor and result in plant retarded growth and senescence was overexpressed.

### Effect of herbicide exposure on tolerant genotype: H[TC][TE]

We found that a large group of transcripts was associated with hydrolase and transferase activity under this category. Additionally, we identified approximately 100 genes for transporter activity and nine related to a structural component of the ribosome (Supplementary Figure 4). Analysis of GO term biological process, we identified more than 225 genes, and approximately 70 genes for protein and nucleic acid metabolic process, respectively. The KEGG pathway analysis suggested that out of 2,139 differentially expressed transcripts, 18 genes encode for amino acid biosynthesis, two genes for *CYP450*, and one gene encodes for *ABC transporter* genes. We also found three and fifty genes that control DNA replication and hormone signaling process, respectively. Out of 2,139 transcripts, 576 exclusively changed their expression after herbicide exposures in tolerant genotypes. We observed that glutathione gene related to xenobiotic metabolism was highly expressed in herbicide-exposed genotype. However, genes involved in the photosynthetic pathway were down-regulated after herbicide exposure. Higher expression of genes involved in DNA replication process in tolerant genotype suggested the indirect role of herbicide on replication process.

In order to predict the effect of herbicide on tolerant plants, we used gene correlation expression analysis on differentially expressed genes at 5-fold change logCPM ≥ 5, FDR ≤ 0.05. Analysis of 33 genes (Additional Files 5, 6) resulted in the identification of a central hub gene based on highest degree which encode for thaumatin-like protein-1 involved in resistance (Punja, 2005). This gene is found to be positively co-expressed with 11 genes and negatively co-expressed with two genes. Out of these 11 genes, 3 were found be related to xenobiotic metabolism and one for disease resistance.

### Gene regulatory network

Various studies have reported that large number of DEGs can be further narrow down by constructing GRN (Hur et al., 2015). It has also been reported that SNPs of genes involved in GRN lead to perturbation of gene regulation affecting phenotype/trait (Kim et al., 2014a) leads to eQTL discovery. In our three GRN model, we have found 141, 68, and 13 genes which are interacting with their respective hub genes are expected to have similar protein-protein interaction model. The magnitude of genes in our GRNs is in the range, which has been reported with a correlation of a particular trait such as 39 genes controlling immunity trait in human (Cai et al., 2013). Since no information is available in public domain, thus “logical model” of GRN was constructed which is based on limited sample size (Vijesh et al., 2013). It reflects the qualitative aspect of genes involved rather than quantitative relationship among the genes in the model as base line information.

Identifying genes responsible for herbicide tolerant cannot absolutely replace herbicide at the moment. Such genes can be used in variety development. Transfer of such resistant genes can minimize the use of herbicide dose. Such resistant gene refinement has been reported in the various crop for example EPSP synthase in soybean, cotton and corn (Funke et al., 2006), AHAS1 gene in sunflower (Bulos et al., 2013), MCPA genes in *Raphanus* (Jugulam et al., 2014) and opaque2 gene in Maize (Danson et al., 2006).

### Marker discovery

Out of 30,803 transcripts, we found only 2,988 transcripts have total of 3,540 SSRs along with motif type and its abundance as shown in Table 6. Tri-nucleotide repeats were found to be most abundant (60.65%) followed by di- (18.92%), mono- (17.15%), hexa- (1.78%), tetra- (1.13%), and penta-nucleotides (0.37%). However, only 885 SSR motifs had been identified in 742 (~12.5%) differentially expressed (DE) genes out of 6,310 total genes. Primer3 software was used to design primers for 1014 SSR motifs successfully (Additional File 7).

**Table 6:** SSR markers motif in Chickpea transcriptome.

**Repeats Type**	**Number of SSRs in Total transcripts**	**Number of SSRs in unique DEGs**
Mono	607 (17.15%)	148 (16.72%)
Di-nucleotide	670 (18.92%)	160 (18.08%)
Tri-nucleotide	2,147 (60.65%)	550 (62.15%)
Tetra-nucleotide	40 (1.13%)	11 (1.24%)
Penta-nucleotide	13 (0.37%)	3 (0.34%)
Hexa-nucleotide	63 (1.78%)	13 (1.47%)

### Identification of SNPs and indels

**Figure 2:**
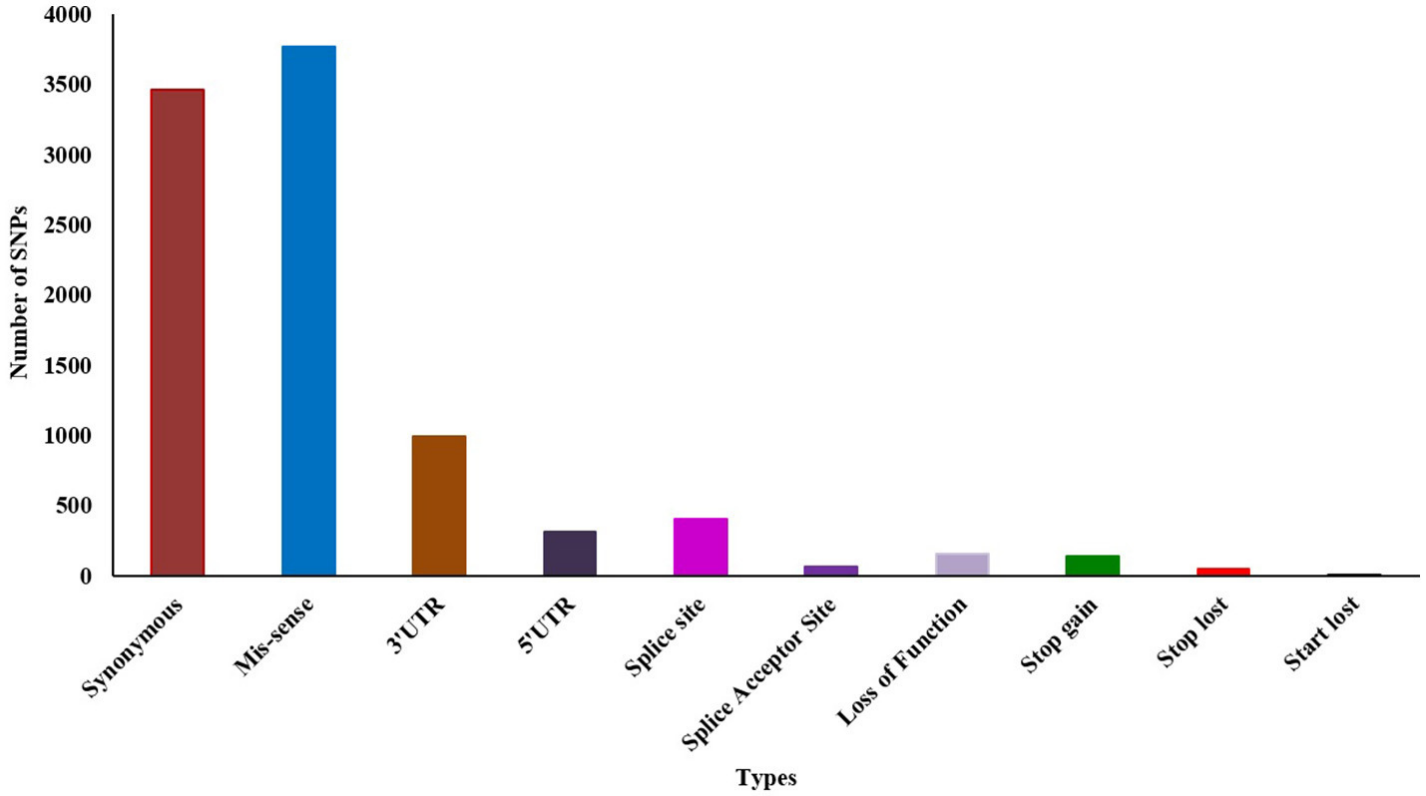
Effect of SNPs in susceptible vs. tolerant genotypes.

Initially, we predicted 14,952 (13,778 SNPs and 1,174 Indel) variations using Samtools from the whole transcript assembly. Further filtering resulted in 9,691 (8,862 SNPs and 829 Indel) highly significant variations at *p*-value 0.05 and MQ ≥ 40. Among the 8,862 SNPs, 5,427 (61.24%) were reported as transition and 3,435 (38.76%) as transversion. Analysis of SNPs' effect showed that 3,464 (39%) and 3,770 (42.54%) are synonymous and missense SNPs, respectively (Additional File 8). We also identified ~1,000 and ~300 SNP's fall in the 3′ and 5′ untranslated region (UTR), respectively (Figure 2). We further evaluated the presence of previously identified 2151 SNPs and observed that 97 (4.5%) were present in our dataset, thus 2,054 (95.5%) are novel.

Findings of the present investigation in terms of candidate genes, transcriptional factors, hub proteins genes associated with herbicide tolerance may be evaluated for direct refinement through Marker Assisted Selection (MAS). Since, more than 80K SSR markers with an approximate marker density of 9 KB are already reported in chickpea across chromosomes (Varshney et al., 2013), thus strategy of MAS can easily be formulated using flanking region markers having polymorphic allele among “donor” and “recipient” genotypes. Such refinement of candidate genes is associated with herbicide tolerance e.g., *Ahasl1* gene in sunflower is already reported using markers viz., SSR, CAPS, and SNP (Bulos et al., 2013) and *opaque2* gene in maize (Danson et al., 2006).

Our enlisted DEGs of four sets namely, 3,537, 1,487, 2,139, and 1,596 (Additional File 2) can be a valuable genomic resource for SNP discovery and future association studies. Uncommon DEGs of susceptible and tolerant genotypes can be further prioritized for SNP discovery and association studies depicting desirable and non-desirable alleles. Such DEG based SNP discovery has been reported in other crops like wheat with cold tolerance (Laudencia-Chingcuanco et al., 2011), dormancy (Barrero et al., 2015), *Brassica* (Devisetty et al., 2014), switchgrass for rust resistance (Serba et al., 2015), white pine (Liu et al., 2014). Similarly, such susceptible/tolerant genotype transcriptome-based approach for eQTL discovery for high-density map has also been reported in *Brassica rapa* (Devisetty et al., 2014). Genes listed in GRN has also been a preferred source of SNP discovery (Kim et al., 2014b) which can be further used in association studies, such approach has been reported in herbaceous model fuel crop, Switch grass (Lu et al., 2013).

## Experimental validation of differential expression data by qRT-PCR

**Figure 3:**
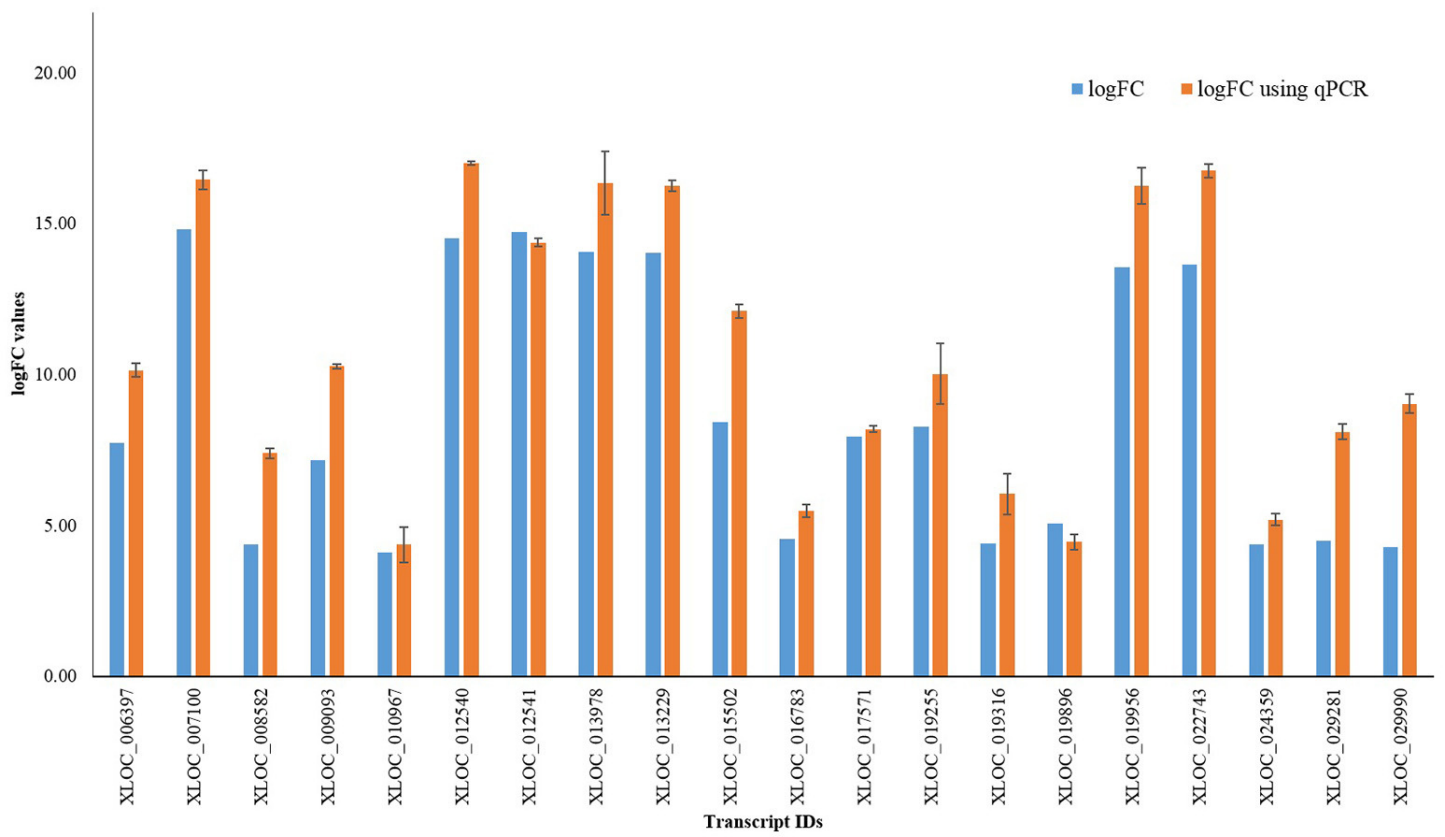
qRT-PCR results of randomly selected 20 transcripts.

In order to validate the magnitude of differential gene expression obtained by transcriptomic approach, qRT-PCR analysis (Additional File 9) was done using randomly selected 20 transcripts. Result obtained was found to be largely corresponding with log fold change value of DEGs (Figure 3).

## Conclusion

This is the first report based on transcriptome studies deciphering genes and its key pathways involved in herbicide response in chickpea crop using susceptible and tolerant genotypes. We report DEGs which are regulated by various miRNAs, TFs, GRN with hub proteins, genic region putative markers like SSRs, SNP and Indels. Differential expression of genes were validated using qPCR. Reported putative candidate genes like glutathione S-transferase (GST), cytochrome P450, xyloglucan endotransglucosylase/hydrolase, glutamate dehydrogenase, methyl crotonoyl carboxylase and thaumatin can be used for further investigation and association studies. Such findings are of immense use in variety improvement programme. Looking at climate change with advent of increase in biotic stress of legume crops, cost of weeding through manual, mechanical, chemical techniques, there is need of weedicide tolerant legume varieties. There is also a hard pressed need to obviate bio-magnification of herbicide in food produce/food-chain of an ecosystem. In endeavor of improving productivity of chickpea germplasm without compromising environmental sustainability more such studies are warranted.

## Author contributions

DK and SC conceived theme of the study. KS, PG, PS, and KA collected sample. DS, MI, SJ, and RJ did the computational analysis of generated data. MI, SJ, KS, and DK drafted the manuscript. SC, NS, RV, and AR edited the manuscript. All co-authors read and approved the final manuscript.

### Conflict of interest statement

The authors declare that the research was conducted in the absence of any commercial or financial relationships that could be construed as a potential conflict of interest.

## Supplementary material

Supplementary Figure 1 Annotation of differentially expressed genes (DEGs) of HS-Control vs. HT-Control: **(A)** Go-term annotation for biological process. **(B)** Cellular component distribution. **(C)** Molecular function. **(D)** Distribution of blast hit identified species.

Supplementary Figure 2 Annotation of differentially expressed genes (DEGs) of HS-Exposed vs. HT-Exposed: **(A)** Go-term annotation for biological process. **(B)** Cellular component distribution. **(C)** Molecular function. **(D)** Distribution of blast hit identified species.

Supplementary Figure 3 Annotation of differentially expressed genes (DEGs) of HS-Control vs. HS-Exposed: **(A)** Go-term annotation for biological process. **(B)** Cellular component distribution. **(C)** Molecular function. **(D)** Distribution of blast hit identified species.

Supplementary Figure 4 Annotation of differentially expressed genes (DEGs) of HT-Control vs. HT-Exposed: **(A)** Go-term annotation for biological process. **(B)** Cellular component distribution. **(C)** Molecular function. **(D)** Distribution of blast hit identified species.

Additional File 1 Blast hits of contigs obtained from unmapped read assembly.

Additional File 2 Blast hits of Differentially Expressed Genes under different conditions.

Additional File 3 Blast hits of identified transcription factors.

Additional File 4 Blast results of identified miRNA and their targets.

Additional File 5 Hubgenes under different conditions.

Additional File 6 Highly significant DEG under different conditions.

Additional File 7 Putative SSRs and its primers.

Additional File 8 Identified SNPs in transcriptome.

Additional File 9 qRT-PCR analysis results of randomly selected 20 transcripts.

## Availability of supporting data

The datasets supporting the conclusions of this article are available in the NCBI repository with following accessions and hyperlinks but is kept at hold till the publication. These would definitely be made public after publication.

BioProject accession: PRJNA306813

https://submit.ncbi.nlm.nih.gov/subs/bioproject/SUB1254173/overview

BioSample accession: SAMN04364319

https://submit.ncbi.nlm.nih.gov/subs/biosample/SUB1249284/overview

BioSample accession: SAMN04364320

https://submit.ncbi.nlm.nih.gov/subs/biosample/SUB1249277/overview

BioSample accession: SAMN04364321

https://submit.ncbi.nlm.nih.gov/subs/biosample/SUB1249281/overview

BioSample accession: SAMN04364322

https://submit.ncbi.nlm.nih.gov/subs/biosample/SUB1249279/overview

The results/conclusions of this article from above datasets are tabulated in excel as additional files and included within the article (Additional Files 1–9).

Since data is generated in the studies and kept in public domain, thus there is no violation of Fort Lauderdale and Toronto agreements.

## Abbreviations

HT: Herbicide Tolerant
HS: Herbicide Susceptible
DEG: Differentially Expressed Gene
SSR-FDM: Simple Sequence Repeat-Functional Domain Marker
SNP: Single Nucleotide Polymorphism
MAS: Marker Assisted Selection
GMO: Genetically Modified Organism
GM-HRC: Genetically Modified Herbicide-Resistant Crops
AHAS: Acetohydroxyacid Synthase
FDR: False Discovery Rate
eQTLs: Expression Quantitative Trait Loci
PCR: Polymerase Chain Reaction
UTR: Untranslated region
NCBI: National Center for Biotechnology Information.
