# Supplementary figures and images for: Discovery of Putative Herbicide Resistance Genes and Its Regulatory Network in Chickpea Using Transcriptome Sequencing
Source: Front Plant Sci. 2017 Jun 7;8:958. doi: 10.3389/fpls.2017.00958 (PMC5461349; doi:10.3389/fpls.2017.00958)

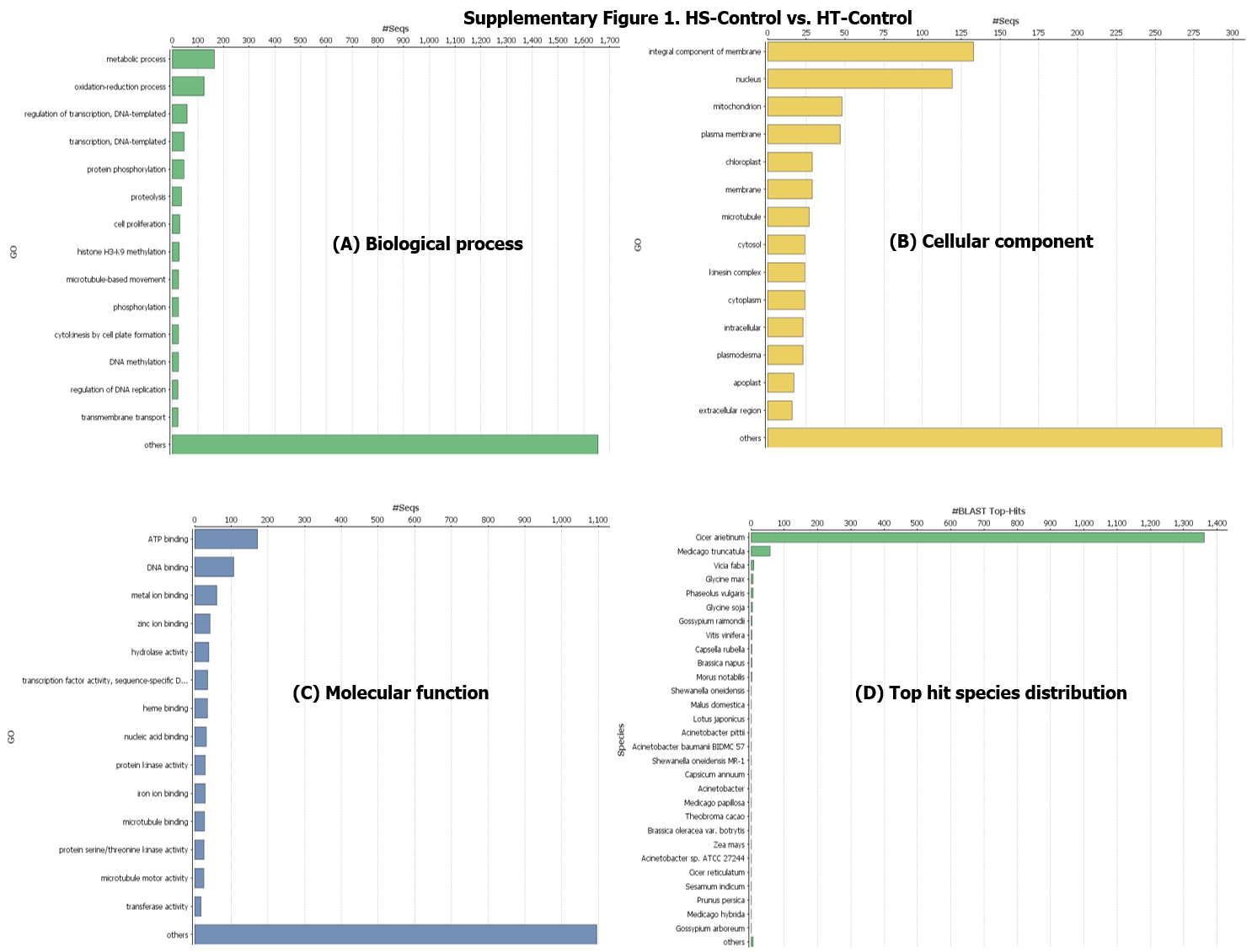

Supplement: Supplementary Figure 1 — Annotation of differentially expressed genes (DEGs) of HS-Control vs. HT-Control: (A) Go-term annotation for biological process. (B) Cellular component distribution. (C) Molecular function. (D) Distribution of blast hit identified species. [file Image1.JPEG]

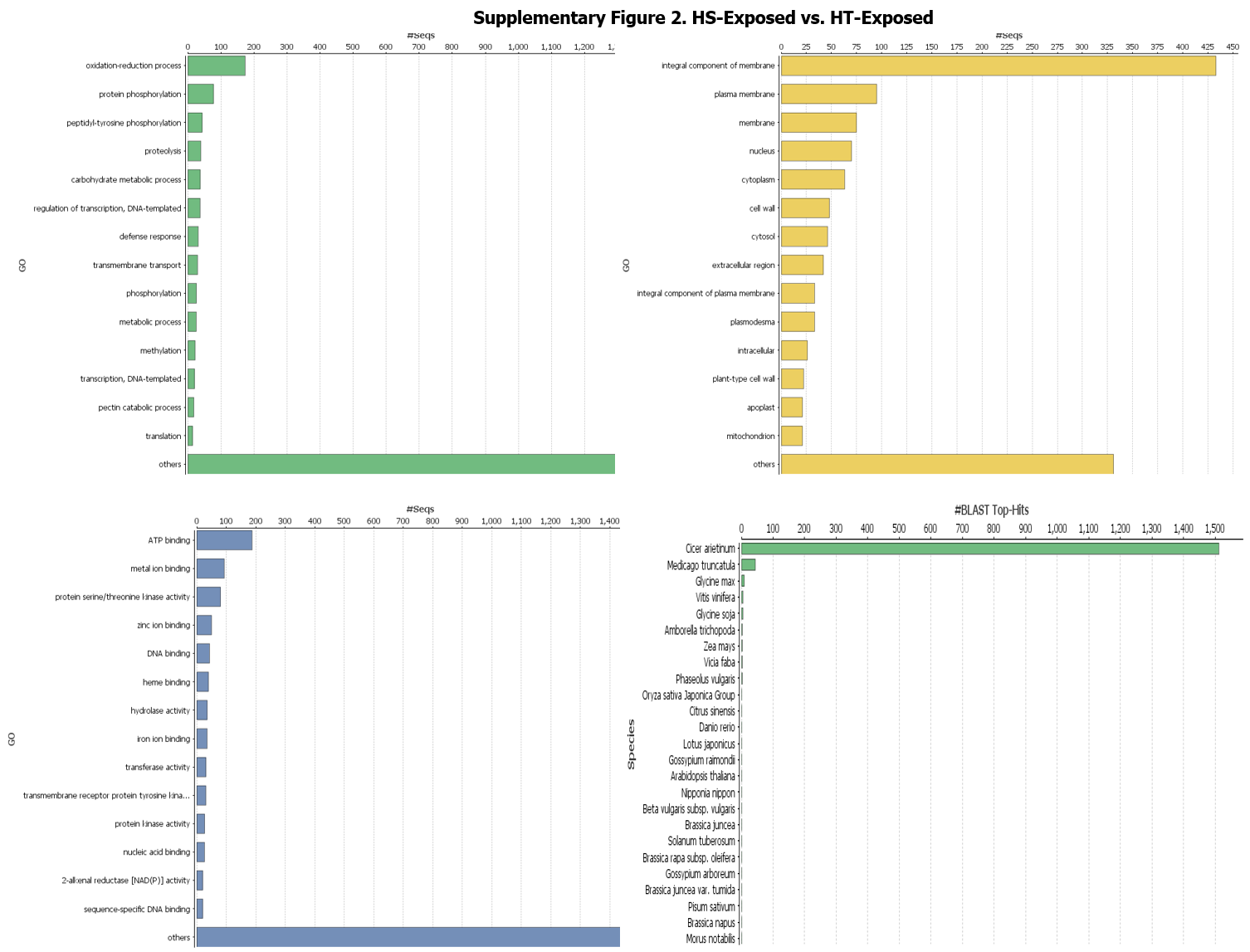

Supplement: Supplementary Figure 2 — Annotation of differentially expressed genes (DEGs) of HS-Exposed vs. HT-Exposed: (A) Go-term annotation for biological process. (B) Cellular component distribution. (C) Molecular function. (D) Distribution of blast hit identified species. [file Image2.JPEG]

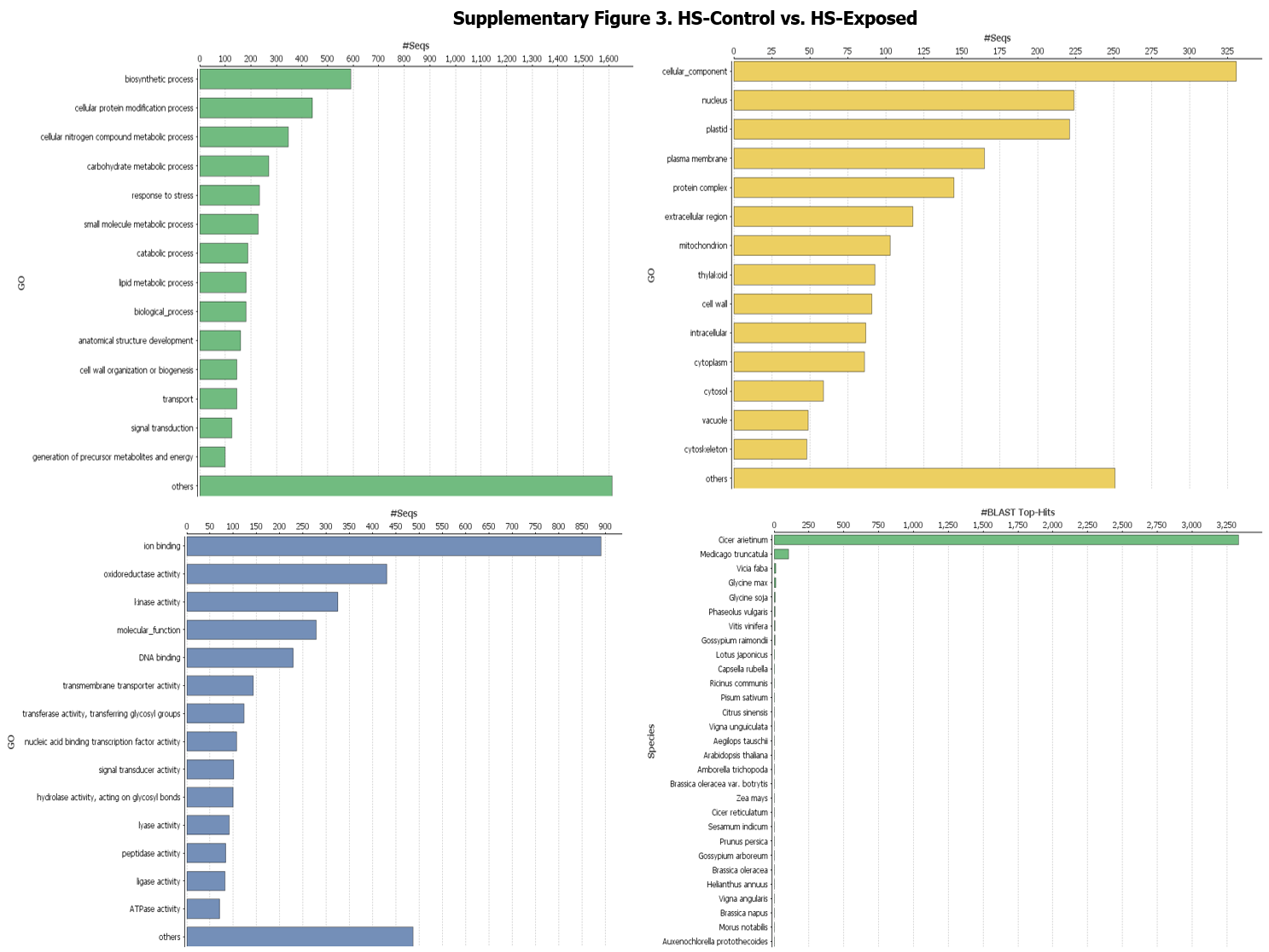

Supplement: Supplementary Figure 3 — Annotation of differentially expressed genes (DEGs) of HS-Control vs. HS-Exposed: (A) Go-term annotation for biological process. (B) Cellular component distribution. (C) Molecular function. (D) Distribution of blast hit identified species. [file Image3.JPEG]

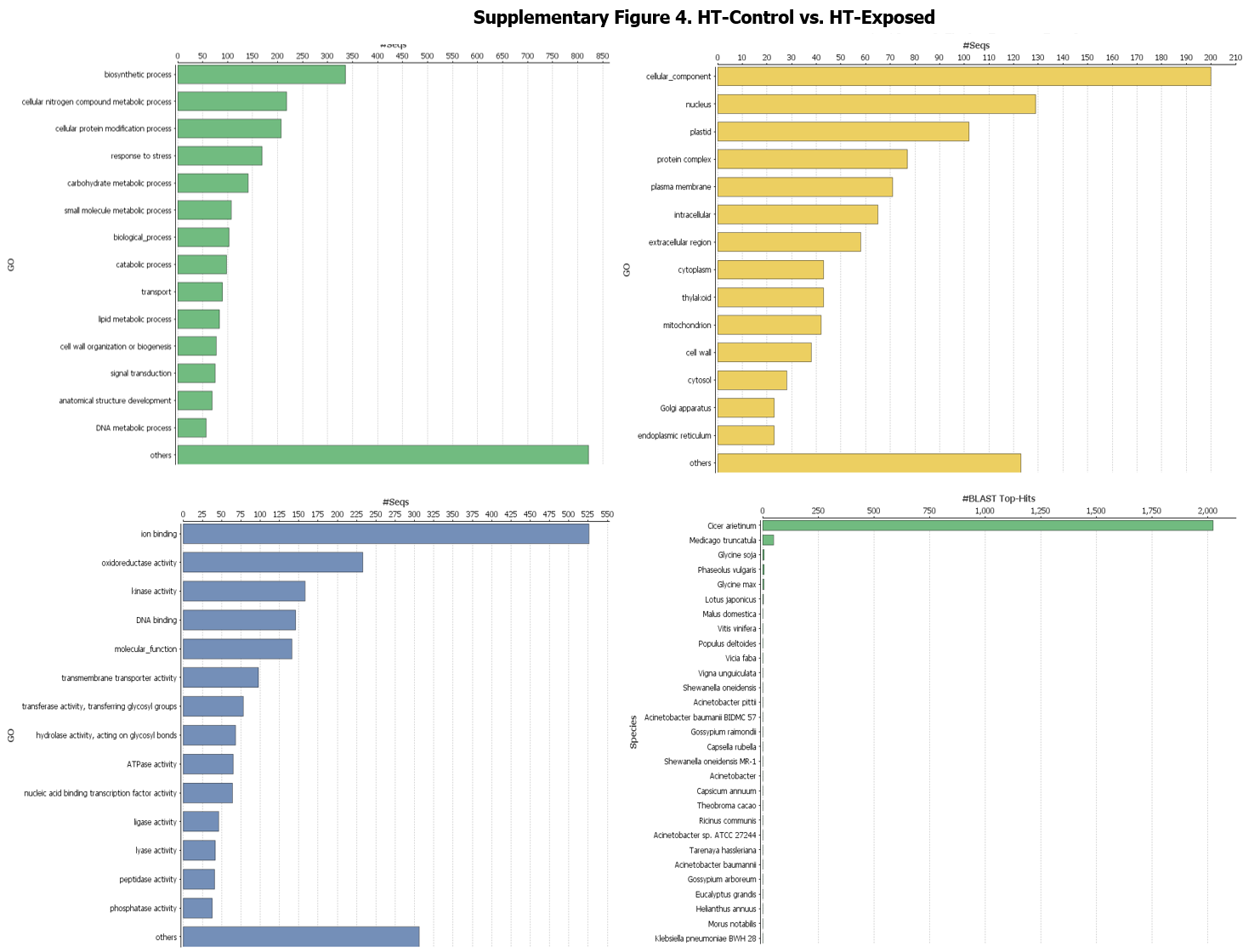

Supplement: Supplementary Figure 4 — Annotation of differentially expressed genes (DEGs) of HT-Control vs. HT-Exposed: (A) Go-term annotation for biological process. (B) Cellular component distribution. (C) Molecular function. (D) Distribution of blast hit identified species. [file Image4.JPEG]
